# Psychotherapeutic approaches to illness anxiety disorder

**DOI:** 10.1192/j.eurpsy.2025.1038

**Published:** 2025-08-26

**Authors:** O. Vasiliu, A. G. Mangalagiu, B. M. Petrescu, C. A. Candea, C. Tudor, D. Ungureanu, M. Miclos, C. Florescu, R. E. Bratu-Bizic, M. Dobre, A. F. Fainarea, M. Patrascu, D. G. Vasiliu

**Affiliations:** 1 Neuroscience Department, “Carol Davila” University of Medicine and Pharmacy; 2 Psychiatry Department, “Dr. Carol Davila” University Emergency Central Military Hospital; 3 White Yellow Cross Foundation, Bucharest, Romania

## Abstract

**Introduction:**

Hypochondriasis was included in DSM-III up to DSM-IV TR in the category of „somatoform disorders,” and ICD-10 concurred with this classification. However, in the last editions of the DSM (i.e., 5 and 5TR), this entity has been transformed into „illness anxiety disorder” (IAD) based on its main clinical feature- the fear of having or contracting a serious disease, although no medical evidence to support such an assumption exists. ICD-11 preserves the term „hypochondriasis” but places it between „obsessive-compulsive or related disorders.” Approaching patients with hypochondria or IAD is reputedly difficult due to the difficulty of maintaining a therapeutic relationship, the sensitivity of these patients to medical data that deny their assumptions of being somatically ill, and their tendency to continuously search the information that validates their belief about their own health.

**Objectives:**

To conduct a literature search to assess the efficacy of psychotherapeutic interventions for patients with IAD.

**Methods:**

This review included three databases (Google Scholar, PubMed, and Web of Science/Clarivate) that were searched from their inception until June 2024 for papers published in English corresponding to the keywords “hypochondriasis,” or illness anxiety disorder,” and “psychotherapy*.”

**Results:**

Cognitive-behavioral therapy (CBT) was used successfully in case reports of IAD when integrated into case management. In a clinical trial, CBT plus fluoxetine led to better results than either intervention alone after 24 weeks. Systematic reviews and meta-analyses showed CBT, cognitive therapy, behavioral therapy and behavioral stress management may significantly reduce hypochondriacal symptoms versus waiting list. The therapeutic approach for IAD was focused on restructuring the catastrophic anticipations, exposure to feared stimuli, and learning relaxation techniques, replacing avoidance and reassurance-seeking with adaptive coping skills and problem-solving techniques. Mindfulness-oriented therapy, group therapies, and acceptance and commitment therapies have also been explored in this population, but the level of quality is low.

**Conclusions:**

CBT remains the only psychotherapy that proved efficacious for patients with IAD, but most of the data retrieved is derived from case reports and small trials. Changes in the terminology and conceptualization of hypochondriasis/IAD may negatively interfere with the possibility of selecting homogenous groups for clinical studies.

**Disclosure of Interest:**

None Declared

